# Pharmacotherapeutic Management of Depression in Patients With Cancer: A Review of Mechanistic and Clinical Evidence

**DOI:** 10.1002/cnr2.70587

**Published:** 2026-05-24

**Authors:** Sepideh Hajivalizadeh, Kimia Farahmand, Kimia Kazemzadeh, Ghazaleh Hajivalizadeh, Ahmad Shamabadi

**Affiliations:** ^1^ Osteoporosis Research Center Endocrinology and Metabolism Clinical Sciences Institute, Tehran University of Medical Sciences Tehran Iran; ^2^ Psychiatric Research Center Roozbeh Psychiatric Hospital, Tehran University of Medical Sciences Tehran Iran

**Keywords:** antidepressant, clinical trial, comorbidity, major depressive disorder, tumor

## Abstract

**Background:**

Depression is a prevalent comorbidity in cancer, yet conventional treatments show inconsistent efficacy. This review sought to provide a mechanism‐based framework for managing cancer‐related depression by exploring its unique pathophysiology and evaluating promising pharmacotherapies based on their alignment with these biological pathways. This narrative review synthesized evidence on promising pharmacotherapies based on their ability to target the core biological mechanisms of cancer‐related depression, including pro‐inflammatory cytokine activity, hypothalamic–pituitary–adrenal axis hyperactivity, serotonin pathway disruption via indolamine‐2,3‐dioxygenase activation, and glutamate excitotoxicity.

**Recent Findings:**

Interventions directly targeting inflammation (e.g., celecoxib) and glutamate modulation (e.g., ketamine) demonstrated encouraging early evidence. The effect of ketamine can also be due to its anti‐inflammatory properties. Atypical antidepressants, such as mirtazapine, and the serotonergic psychedelic psilocybin also showed promising but preliminary benefits. In contrast, conventional selective serotonin reuptake inhibitors and tricyclic antidepressants yielded conflicting results. Other agents, including the psychostimulant methylphenidate, showed utility for specific symptoms like fatigue.

**Conclusion:**

A personalized, mechanism‐informed approach targeting the core pathophysiological cascade of inflammation, hypothalamic–pituitary–adrenal axis hyperactivity, and glutamate excitotoxicity is essential for effectively managing depression in patients with cancer. This represents a necessary paradigm shift away from a one‐size‐fits‐all treatment model.

## 1 Background

Cancer remains a leading global health burden, accounting for one in every six deaths. In 2022, approximately 20 million new cancer cases and 10 million cancer‐related deaths were reported [1]. The diagnosis and treatment of cancer can be a profoundly stressful experience, leading to significant emotional distress and psychological challenges. Anxiety and depressed mood are common reactions in individuals undergoing or having completed cancer treatment [2]. Despite the advancement in survival rates of cancer patients over the recent decades, several patients report dealing with comorbid anxiety or depression. The most prevalent psychological condition among cancer patients is depression. The prevalence of depression is approximately between 8% and 32% among cancer patients. One in every five or six cancer patients experiences clinical depression following a cancer diagnosis during the first year [3, 4]. Research indicates that individuals diagnosed with cancer exhibit a twofold increase in the likelihood of experiencing depression relative to the general population [5].

The concurrent presence of cancer and depression has been extensively documented; however, the exact biological pathways connecting these two conditions have yet to be fully elucidated [6]. Comorbidity of cancer and depression results in 113% higher health care costs compared to non‐depressed patients diagnosed with cancer and worse outcomes, including higher rates of suicide and diminished treatment adherence [4, 7]. These findings underscore the urgent need for effective, evidence‐based management strategies for depression among cancer patients.

Depression treatment in cancer patients encompasses pharmacotherapy and psychotherapy [4]. Conventional pharmacotherapy for depression involves two generations of antidepressants. The first generation includes monoamine oxidase inhibitors and tricyclic antidepressants (TCAs). The second generation includes serotonin‐norepinephrine reuptake inhibitors and selective serotonin reuptake inhibitors (SSRIs) [8]. Regarding depression pharmacotherapy, the choice of medication should be made based on the specific patients' conditions. The risk of overdose, adverse effects, and mortality is higher in TCAs compared to SSRIs. TCAs can also aggravate chemotherapy‐induced delirium. On the other hand, SSRIs can exacerbate nausea and vomiting in patients under treatment with radiotherapy or chemotherapy [4, 9].

Generally, it is reported that at least 30% of individuals diagnosed with depression fail to respond to conventional antidepressant treatments. Comorbidities like cancer can raise the likelihood of treatment‐resistant depression [10]. Moreover, based on the results of a cohort study investigating depression treatment in individuals diagnosed with advanced‐stage cancer, antidepressant treatment did not demonstrate a significant effect on depression scores over time [11].

Previous research has explored some aspects of the link between cancer and depression [12, 13, 14, 15]. Furthermore, prior reviews have investigated the efficacy and options of pharmacotherapy for depression management in oncology settings [13, 15, 16, 17, 18, 19, 20, 21, 22, 23, 24, 25, 26, 27]. However, they lack a comprehensive, mechanistic exploration of both depression pathogenesis and mechanisms of action of diverse antidepressants used for cancer patients. While previous reviews have cataloged treatment options, a comprehensive synthesis linking the unique pathophysiology of cancer‐related depression, driven by inflammation, hypothalamic–pituitary–adrenal (HPA) axis hyperactivity, and glutamate dysregulation, to the specific mechanisms of various pharmacotherapies is needed.

The common focus on monoamine‐based mechanisms often fails to account for the inconsistent efficacy of conventional antidepressants in this population. Several established theories of depression support this mechanism‐based framework, including inflammatory, serotonergic, glutamatergic, and HPA‐axis dysregulation models. The inflammatory hypothesis links immune activation and cytokine signaling to depressive symptoms and reduced antidepressant responsiveness [28]. Dysregulation of the HPA axis, characterized by chronic hypercortisolemia and impaired negative feedback, has also been implicated, particularly in the context of chronic stress and medical illness [29]. The serotonin theory of depression remains influential, although umbrella‐level evidence suggests that it does not fully explain depression in all patients [30]. In parallel, the glutamate hypothesis highlights synaptic dysfunction and altered excitatory neurotransmission as additional contributors to mood symptoms and treatment resistance [31]. Together, these models provide a biologic rationale for evaluating pharmacotherapies that go beyond a purely monoaminergic approach.

This review argues that a mechanism‐based approach is essential for improving therapeutic outcomes. By systematically linking the underlying neurobiology of cancer‐related depression to the pharmacological actions of various agents, this review aims to provide a new framework for evaluating pharmacotherapy and guiding future clinical strategies in psycho‐oncology. Whereas prior reviews have often grouped treatments by symptom profiles (e.g., pain or sleep disturbance), pharmacotherapies were explicitly organized by their alignment with underlying pathways (inflammation, HPA dysregulation, glutamate, etc.) implicated in cancer‐related depression. This approach aims to better align pharmacotherapy with underlying pathophysiological processes. This review seeks to answer the following key questions:

1. What are the primary neurobiological pathways linking cancer pathophysiology and its treatment to the development of depressive symptoms?
2. What are the efficacious pharmacotherapies for depression in cancer patients?
3. Based on the mechanistic alignment, what is the current clinical evidence for their efficacy and safety in the oncology setting, and what are the implications for clinical practice?

## 2 Methods

This narrative review was conducted considering a scale for the quality assessment of narrative review articles (SANRA) [32] to provide a mechanism‐based evaluation of pharmacotherapies for cancer‐related depression. The literature was identified through a structured, two‐stage search designed to first identify promising agents and then to gather comprehensive evidence for each one.

An initial search was conducted in the PubMed database on August 14, 2024, limited to articles classified as Clinical Trial or randomized controlled trial (RCT) with no other restrictions, including language and date, using the query (“major depressive disorder (MDD)” OR depression) AND (cancer OR tumor). Additional databases were not searched. From this pool of high‐level original evidence, medications were selected for inclusion if they were reported in at least one phase II RCT to improve depressive symptoms in patients with cancer. Promising mechanistically fit agents with emerging evidence were also discussed to reflect evolving therapeutic directions. Studies were excluded if they did not report depression‐specific outcomes or were not conducted in cancer populations. Conversely, commonly used antidepressants were not included because they did not meet the predefined criterion of demonstrating efficacy in depression outcomes specifically within cancer populations. The search retrieved 250 records; 21 of which, covering 12 medications, were included based on relevance to depression outcomes in cancer populations.

For each medication identified, a second, broader search was conducted in PubMed to retrieve all relevant literature. This search used the name of the specific medication combined with the initial search string, but without any restrictions on article type. Reference lists of key articles were reviewed to find missed relevant papers. This two‐stage approach allowed for an initial evidence‐based selection of promising medications, followed by a comprehensive gathering of all pertinent data, including clinical trials, reviews, case reports, and preclinical research for the selected agents. The initial filtration step focused the review on agents with a demonstrated signal of efficacy, while the second stage enabled a balanced and critical appraisal of the complete evidence base for these promising candidates.

## 3 Pathophysiology

### 3.1 Biological Mechanisms

#### 3.1.1 Neurobiological Factors

**FIGURE 1:**
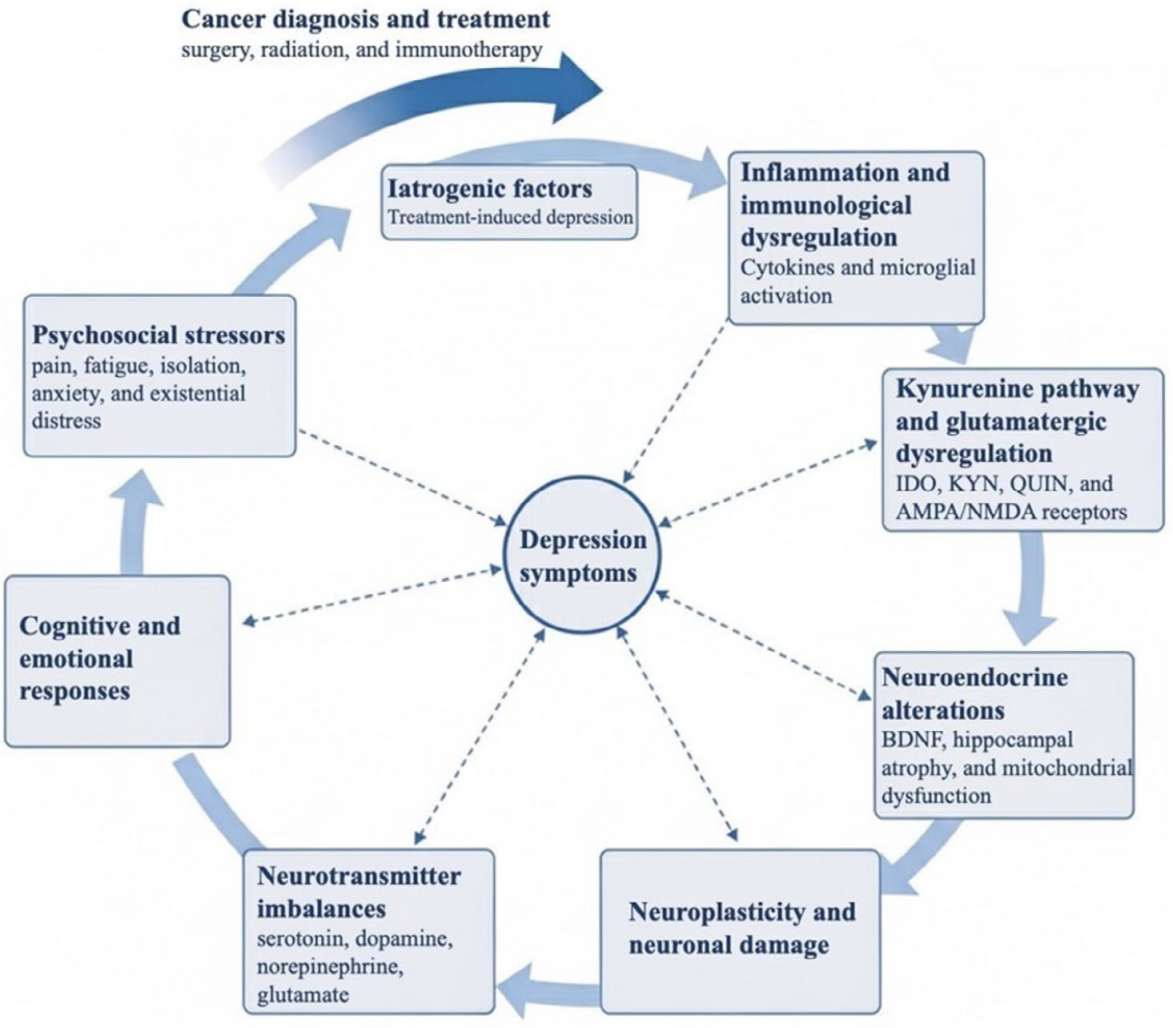
The neuropathophysiology of depression in cancer patients. This diagram illustrates the complex and bidirectional interplay between various biological and psychosocial factors contributing to the development and maintenance of depressive symptoms in cancer patients. Cancer diagnosis and its treatments can directly lead to iatrogenic factors and stimulate systemic inflammation and immunological dysregulation. This dysregulation activates the kynurenine pathway, leading to glutamatergic dysregulation and excitotoxicity, involving enzymes like indoleamine 2,3‐dioxygenase (IDO), and metabolites such as kynurenine (KYN) and quinolinic acid (QUIN), affecting α‐amino‐3‐hydroxy‐5‐methyl‐4‐isoxazolepropionic acid (AMPA) and N‐methyl‐D‐aspartate (NMDA) receptors. These biological changes, alongside psychosocial stressors, trigger neuroendocrine alterations (e.g., hypothalamic–pituitary–adrenal axis dysfunction, hippocampal atrophy, mitochondrial dysfunction) and impact neurotransmitter imbalances. These interconnected pathways culminate in neuroplasticity and neuronal damage, ultimately manifesting as depression symptoms. Furthermore, cognitive and emotional responses to these stressors can create a vicious cycle, exacerbating psychosocial burdens and influencing biological pathways.

The neurobiological link between cancer and depression may be understood from an evolutionary perspective. Several theories attempt to explain how cancer biology may contribute to the onset of depression, focusing on inflammatory mediators, an overactive HPA axis, and changes in neurotransmitter concentrations. The crosstalk between inflammatory pathways and neurocircuits in the brain evolved to produce adaptive sickness behaviors in response to pathogens. These behaviors conferred a survival advantage by helping organisms fight infection. In modern times, however, this same system can be chronically and pathologically activated by non‐infectious threats, such as the physiological and psychological stress of cancer and its treatment. This sustained inflammatory response appears to drive the development of depressive syndromes and may contribute to the non‐responsiveness often seen with conventional antidepressant therapies. This framework positions inflammation not as a mere correlate of depression, but as a central driver of its pathophysiology in the context of cancer [6, 33]. Figure 1 shows a general schematic of the neurobiological mechanisms of depression in patients with cancer.

Depression is associated with changes in the levels and function of several key neurotransmitters, including serotonin (5‐HT), norepinephrine, and dopamine [34, 35]. Reduced levels of 5‐HT are linked to changes in mood, appetite, sleep, sex drive, pain response, body temperature, and circadian rhythm. Some tumors can disrupt serotonin synthesis, either by increasing the local concentration of serotonin or its metabolites or by decreasing serotonin reserves in the brain, which can lead to depressive symptoms. On the other hand, an increase in 5‐HT can stimulate the PI3K‐Akt–mTOR pathway, promoting tumor metabolism and growth while simultaneously reducing serotonin levels in the brain [34].

Pro‐inflammatory cytokines may also disrupt serotonin synthesis [9, 34]. Specifically, the activity of the enzyme indolamine‐2,3‐dioxygenase (IDO) increases due to pro‐inflammatory cytokines, like tumor necrosis factor (TNF)‐α, which degrades tryptophan, the chemical precursor to 5‐HT, resulting in reduced 5‐HT levels [9]. IDO catalyzes the conversion of tryptophan, the primary amino acid precursor of serotonin, into kynurenine (KYN). This process results in two major consequences: first, a decrease in tryptophan levels leads to serotonin deprivation; second, activation of the KYN pathway produces neurotoxic metabolites that can adversely affect mood and cognitive function.

KYN can be converted into either kynurenic acid (KYNA) or quinolinic acid (QUIN), depending on the cell type involved. In astrocytes, KYN is typically converted to KYNA, which exhibits neuroprotective properties by inhibiting glutamate release and antagonizing N‐methyl‐D‐aspartate (NMDA) receptors. Conversely, in microglia, KYN is preferentially converted to QUIN, a potent NMDA receptor agonist that leads to excessive glutamate release and oxidative stress. This cascade of events contributes to neurodegeneration and excitotoxicity associated with depression [36, 37].

It was reported that stress and inflammation can activate microglia, which may exacerbate neurodegenerative pathways linked to QUIN. This activation is thought to correlate with decreases in brain volume observed in chronic depression patients. The interplay between these mechanisms underscores the complex relationship between cancer‐related inflammation and mood disorders [38].

In an animal model developed by Moreau et al. [39], chronic inflammation was induced using Bacillus Calmette‐Guerin, leading to an initial presentation of sickness behaviors that later evolved into sustained depressive‐like symptoms. This study highlights a potential link between inflammation, IDO activation, and depression within the cancer context. As such, the dysregulation of serotonin synthesis through IDO activation not only contributes to mood disorders but may also influence cancer progression and metastasis [40].

The HPA axis is a crucial component of the body's stress response system. The hypothalamus, pituitary gland, and adrenal glands work together to mediate stimuli when stressors threaten homeostasis. Cytokines, such as TNF‐α, interferon (IFN)‐α, and IFN‐γ, can stimulate the HPA axis [6]. Chronic stress can cause the HPA axis to become dysregulated, which has negative consequences for the body [6, 41].

Depression and stress are positively correlated, where depression can be caused or precipitated by stress [41]. Hyperactivity of the HPA axis is a characteristic feature of MDD. Chronic activation of the HPA axis can lead to dysregulation, potentially manifesting as anxiety and depression. The immune system's activity in cancer can also be linked to the activation of the HPA axis. The HPA axis operates through a negative feedback loop, where glucocorticoids, released due to HPA axis activity, bind to glucocorticoid receptors (GRs) to shut off the cycle [6]. Mineralocorticoid receptors have a high affinity for corticosteroids, while GRs have a low affinity for endogenous steroid hormones [6, 29]. During a stress response, when the concentrations of these substances are higher than basal levels, GRs are more important than mineralocorticoid receptors for regulation. Negative feedback occurs at the hypothalamus, on corticotropin‐releasing hormone (CRH) secretion, and at the pituitary, on adrenocorticotropic hormone (ACTH) release. The hippocampus may also act as a regulatory site due to its density of GRs [6].

Patients with depressive disorders consistently exhibit hyperactivation of the HPA axis, despite these regulatory mechanisms [6]. This hyperactivity impairs the immune response and may contribute to the development and progression of certain types of cancer [41]. It was claimed that HPA axis hyperactivity and elevated inflammation are key pathophysiological mechanisms underlying depression [42]. Various forms of HPA axis dysregulation have been reported in people with cancer, with most studies indicating increased baseline cortisol levels [43]. Impaired GR function is crucial for HPA axis hyperactivity in depression. Antidepressant treatment has been shown to increase GR expression, function, and GR‐mediated HPA axis feedback inhibition, reducing resting and stimulated HPA axis activity. Normalization of GR function by antidepressant treatment is a significant predictor of long‐term clinical outcomes [29].

Experimental models of decreased GR expression have been shown to lead to depressive‐like behavior. Pro‐inflammatory cytokines can reduce GR function [29]. The HPA axis also exhibits circadian and ultradian rhythmicity, with ACTH and corticosteroids secreted in a pulsatile manner. CRH initiates this oscillatory activity, but it is a function of a sub‐hypothalamic pulse generator that produces oscillations in ACTH and glucocorticoids through activation of the feedforward feedback interactions of the pituitary–adrenal peripheral network [44].

Glutamate, the primary excitatory neurotransmitter in the central nervous system, plays a pivotal role in synaptic plasticity, learning, and memory. However, its dysregulation can lead to excitotoxicity, a pathological process characterized by excessive activation of glutamate receptors, particularly the NMDA receptors. This phenomenon is increasingly recognized as a significant contributor to mood disorders, including MDD [45]. Under normal physiological conditions, glutamate levels in the extracellular space are tightly regulated to maintain optimal neuronal function. Astrocytes, a type of glial cell, are crucial for this regulation as they express excitatory amino acid transporters that uptake excess glutamate from the synaptic cleft and convert it into glutamine via glutamine synthetase [46, 47]. However, pathological conditions such as inflammation or chronic stress can impair this regulatory mechanism [48].

In cases of neuroinflammation, often seen in cancer patients, pro‐inflammatory cytokines can disrupt the normal functioning of astrocytes. For instance, TNF‐α can increase the expression of glutaminase in astrocytes, which converts glutamine back to glutamate. This counteracts the astrocytes' role in maintaining glutamate homeostasis and can exacerbate excitotoxicity by elevating extracellular glutamate levels [49, 50]. When extracellular glutamate concentrations rise significantly, potentially exceeding 100‐fold, the risk of excitotoxicity increases. This excessive activation of NMDA receptors leads to a cascade of intracellular events that culminate in neuronal injury and death. The overactivation results in increased calcium influx into neurons, triggering pathways associated with oxidative stress and mitochondrial dysfunction, ultimately leading to cell apoptosis [45, 51].

The KYN pathway is another critical aspect contributing to glutamate dysregulation and excitotoxicity. In conditions where IDO is activated, such as during cancer or chronic inflammation, tryptophan is diverted from serotonin synthesis toward KYN production. As previously mentioned, KYN can be converted into neurotoxic metabolites like QUIN, which acts as an NMDA receptor agonist. Elevated QUIN levels can further enhance glutamatergic transmission and contribute to excitotoxic processes [49, 52].

Conversely, KYN can also be converted into KYNA, which has neuroprotective effects by antagonizing NMDA receptors and inhibiting excessive glutamate release. The balance between QUIN and KYNA production is crucial; an imbalance favoring QUIN may exacerbate depressive symptoms through enhanced excitotoxicity and neuronal damage [51, 52].

#### 3.1.2 Inflammatory Processes

Cytokines play a crucial role in the relationship between inflammation and depression, particularly in cancer patients. Pro‐inflammatory cytokines such as interleukin (IL)‐6 and TNF‐α have been consistently linked to depressive symptoms. Elevated levels of these cytokines are often observed in patients with cancer who experience depression, suggesting a biological underpinning for their psychological distress [9, 53]. Production of these cytokines can be triggered by treatment modalities like chemotherapy and radiation, which induce tissue damage and subsequent inflammatory responses [9, 54].

The mechanisms by which these cytokines contribute to depression are multifaceted. For instance, TNF‐α and IL‐6 can enhance the activity of serotonin and norepinephrine reuptake transporters through pathways involving p38 mitogen‐activated protein kinase, leading to decreased availability of these neurotransmitters in the synaptic cleft [9, 54]. This reduction is significant because serotonin and norepinephrine are critical for mood regulation. Furthermore, pro‐inflammatory cytokines may also increase the secretion of CRH, which has been implicated in the behavioral changes associated with depression [9, 55]. For example, among 203 patients with glioma, 66.5% showed depressive symptoms, and IL‐6 and TNF‐α showed good performance in accurately predicting depression in these patients [56].

Bouchard et al. discovered that pro‐inflammatory cytokines, including IL‐1β and TNF‐α, are positively correlated with depressed mood in patients with breast cancer [57]. Similarly, McFarland et al. found that depressed patients with lung cancer had higher levels of pro‐inflammatory cytokines compared to patients without depressive symptoms [58]. Du et al. demonstrated that sputum IL‐6 and TNF‐α served as effective predictors for depressive symptoms in patients with lung cancer [59].

The concept of sickness behavior provides an important framework for understanding how inflammation can lead to depressive symptoms. This hypothesis posits that the body's immune response to infection or injury, characterized by behaviors such as fatigue, social withdrawal, and decreased appetite, can mimic depressive states [6, 55]. These behaviors are thought to be adaptive responses aimed at conserving energy and promoting recovery. However, in cancer patients, chronic activation of this immune response can lead to sustained depressive symptoms, as the overlap between sickness behavior and clinical depression becomes increasingly pronounced [53, 60].

These observations also support the potential role of anti‐inflammatory treatments, which are further discussed in subsequent sections. Indeed, meta‐analyses show that anti‐inflammatory treatments, particularly cyclooxygenase (COX)‐2 inhibitors like celecoxib, significantly reduce depressive symptoms. For example, Köhler et al. [61] found that add‐on celecoxib at 400 mg/day led to significantly greater symptom improvement than placebo.

#### 3.1.3 Endocrine Changes

The intricate relationship between cancer, stress, and mental health involves several biological mechanisms, including endocrine changes [9]. Stressful conditions arising from a cancer diagnosis can disrupt the HPA axis. Chronic stress stimulates the release of CRH from the hypothalamus, which in turn triggers the secretion of ACTH from the pituitary gland. ACTH then prompts the adrenal cortex to release cortisol into the bloodstream [62].

Studies measuring IL‐6 levels and relative diurnal cortisol variation in depressed cancer patients have revealed that IL‐6 levels are significantly increased, while relative diurnal cortisol variation is decreased among cancer patients with depression compared to those without depression. Screening tests for depression in cancer patients using diurnal cortisol variation may be useful in detecting depression in cancer patients [9]. Furthermore, patients with metastatic breast cancer who exhibit higher average diurnal cortisol concentrations and depressive symptoms, indicative of chronic stress, often show suppressed cell‐mediated immunity [63]. Proinflammatory cytokines also reduce levels of neural growth factors like brain‐derived neurotrophic factor, which is key for neurogenesis, and low levels of brain‐derived neurotrophic factor and neurogenesis have been implicated in the pathogenesis of depression [9].

Crucially, these neurobiological factors are not independent but form an interconnected cascade initiated by inflammation. Cancer‐related inflammation, driven by pro‐inflammatory cytokines such as TNF‐α and IL‐6, acts as the central upstream driver. These cytokines simultaneously induce HPA axis hyperactivity while impairing GR negative feedback, and activate the IDO enzyme. IDO activation not only depletes the essential serotonin precursor, tryptophan, but also shunts its metabolism down the KYN pathway, generating the neurotoxic NMDA receptor agonist QUIN. This directly promotes the glutamate excitotoxicity detailed previously. Thus, a single pathological process, that is, chronic inflammation, triggers a triad of serotonergic disruption, HPA axis dysregulation, and glutamatergic over‐activation, creating a robust and multifaceted biological substrate for depression in cancer patients.

### 3.2 Psychological Mechanisms

Depression in cancer patients is a multifaceted issue influenced not only by biological factors but also significantly by psychological and social dynamics. The emotional and cognitive responses to a cancer diagnosis, coupled with the impact of social factors, play a crucial role in the development and experience of depression among cancer patients [9].

#### 3.2.1 Emotional and Cognitive Responses

A cancer diagnosis is a life‐altering event that can trigger a range of intense emotional responses. While some sadness is a normal reaction, the stress of a cancer diagnosis can overwhelm a person's coping mechanisms and lead to MDD. High levels of mental distress over extended periods can result in anxiety, depression, or a combination of both. These psychological challenges significantly affect a patient's quality of life and overall prognosis [9]. Cancer patients often grapple with fears of death, the disruption of life plans, and changes in their body image and self‐esteem [64]. The uncertainty surrounding treatment outcomes, the possibility of recurrence, and the impact on their future can lead to existential concerns and a sense of hopelessness [9]. These emotional and existential stressors contribute significantly to the development of depression [64].

Individuals respond to a cancer diagnosis and treatment in various ways, employing different coping mechanisms to manage their emotional distress [65]. Adaptive coping strategies, such as strong emotional support from family and friends and maintaining an optimistic outlook, can protect against depression. Conversely, maladaptive coping strategies and poor communication with medical practitioners increase the risk of developing depression [9]. Psychological adaptive mechanism maturity and older age can predict lower depression mood symptoms, meaning those with less mature coping mechanisms are more prone to depressive symptoms [65].

#### 3.2.2 Social Factors

Social factors significantly influence the mental well‐being of cancer patients [66]. A lack of social or family support, along with loneliness, social isolation, and stressful life events, is associated with a higher risk of depression and anxiety among cancer patients. Strong emotional support from friends and family can buffer the negative effects of cancer‐related stress and improve overall quality of life [67].

Also, cancer and its treatments can strain interpersonal relationships and disrupt social roles. Changes in physical appearance, abilities, independence, and finances can affect a patient's social identity and self‐esteem, potentially leading to feelings of isolation and alienation. Difficulties in maintaining social connections and fulfilling roles can contribute to depression [67, 68].

In addition, sociodemographic characteristics such as age, sex, marital status, and income level can also influence the risk of depression [66, 68]. Notably, male cancer patients were found to be less likely to be depressed compared to female patients [67]. Cancer patients with a family history of depression were almost six times more likely to be depressed [67].

Additionally, developmental trauma is an important vulnerability factor. For instance, Montague et al. found that cancer patients with ≥ 3 adverse childhood experiences had a 3.82‐fold higher odds of psychotropic medication use compared to those with ≤ 2 adverse childhood experiences, indicating that early‐life trauma is linked to depression burden and treatment needs in oncology settings [69].

### 3.3 Iatrogenic Factors

Iatrogenic factors encompass the adverse effects of medical treatments and interventions, which can inadvertently contribute to the onset or exacerbation of depressive symptoms [9].

#### 3.3.1 Treatment‐Induced Depression

Cancer treatments, while essential for combating the disease, often carry a range of side effects that can significantly impact a patient's mental health. Chemotherapy, radiation, and other systemic therapies can induce depression through various mechanisms. The physical side effects of cancer treatments can be debilitating and significantly contribute to depression. For instance, chemotherapy and radiation can cause fatigue, pain, nausea, and other physical symptoms that diminish a patient's quality of life and overall well‐being [55]. These symptoms can lead to a sense of helplessness and hopelessness, increasing the risk of depression [9].

In addition to the physical side effects, cancer treatments can also have a profound psychological impact on patients. Treatment‐related changes in body image, such as hair loss, skin changes, or weight fluctuations, can lead to feelings of self‐consciousness, shame, and social isolation [70]. These changes can negatively affect a patient's self‐esteem and sense of identity, contributing to depression [9]. Furthermore, cancer treatments can disrupt a patient's daily routine, social activities, and overall lifestyle. The loss of independence and the inability to participate in activities they once enjoyed can lead to feelings of frustration, sadness, and loneliness, further increasing the risk of depression [55].

Specific medications, such as the dopamine D2 receptor antagonist haloperidol (used to treat chemotherapy‐associated nausea), can reduce dopaminergic transmission in the brain and have been linked to the development of depressive symptoms [70]. Immunotherapy agents, including IFN‐α, have been reported to cause depression in up to 50% of patients [9]. Steroids and androgen deprivation therapy can also induce depression. Iatrogenic distress, arising from inadequate communication, insufficient attention to psychological concerns, and fragmented care, may contribute to an increased risk of depression, anxiety, and post‐traumatic stress disorder [70].

## 4 Medications

Several pharmacotherapeutic options have been reported to manage depression in patients with cancer. The pharmacotherapeutic agents discussed in this section were selected based on a comprehensive survey of the psycho‐oncology literature to provide a mechanism‐based evaluation of relevant treatments. To focus the review on agents with a notable signal of efficacy, those investigated in RCTs for depression in cancer patients were prioritized. This initial step ensured that the core of the discussion was grounded in high‐level clinical evidence. For each agent selected through this process, a subsequent, broader literature search was conducted to retrieve a representative body of evidence. This second stage enabled a more critical appraisal of the complete evidence base, including conflicting, negative, and supportive findings for the most promising therapeutic candidates, thereby providing a balanced and clinically relevant overview for clinicians and researchers. This approach allows for a critical discussion that connects each agent's pharmacological mechanism of action to the underlying neurobiology of cancer‐related depression outlined in the previous section, thereby facilitating a nuanced, mechanism‐informed analysis.

Finally, 12 medications were identified for inclusion in this review; Table 1 presents a summary of the data on these medications reported in the selected studies. Although no age criterion was applied for the inclusion, all patient populations discussed are adults. For consistency, each medication is described using a standardized structure, including (i) mechanism of action, (ii) study characteristics (sample size, cancer type, dose, duration), (iii) antidepressant outcomes, and (iv) safety considerations.

**TABLE 1:** The characteristics of the included clinical trials.

Medication	Mechanism of action	First author/publication year	Sample size	Patients' age	Intervention groups	Daily dosage of the medication	Depression assessment tool	Superiority over the control group
Fluoxetine	Serotonergic system: SSRI, increases serotonin availability by inhibiting reuptake [71, 72].	Fisch/2003 [73]	129	Not reported	Fluoxetine/placebo	20 mg	BZSDS	Yes
		Razavi/1996 [74]	91	≥ 18	Fluoxetine/placebo	20 mg	HADS	No
		Holland/1998 [75]	40	Not reported	Fluoxetine/desipramine	20–40 mg	HDRS	Yes
Paroxetine	Serotonergic system: SSRI, increases serotonin availability by inhibiting reuptake [76, 77].	Roscoe/2005 [78]	94	31–79	Paroxetine/placebo	20 mg	CES‐D/POMS‐DD	Yes
		Pezzella/2001 [79]	175	18–65	Paroxetine/amitriptyline	20–40 mg	MADRS	Yes
		Musselman/2006 [80]	35	18–75	Paroxetine/desipramine/placebo	20–40 mg	HDRS	No
Celecoxib	Inflammatory system: COX‐2 inhibitor, reduces neuroinflammation linked to depressive symptoms [81].	Alamdarsaravi/2017 [82]	81	18–65	Celecoxib/placebo	400 mg	HDRS	Yes
		Mohammadinejad/2015 [83]	52	18–70	Celecoxib/diclofenac	400 mg	HDRS	Yes
Mirtazapine	Serotonergic and noradrenergic systems: Alpha‐2 adrenergic antagonist, blocks 5‐HT2 and 5‐HT3 receptors [84, 85].	Cankurtaran/2008 [86]	53	18–65	Mirtazapine/imipramine/control	5–30 mg	HADS	Yes
		Kim/2008 [87]	42	22–79	Mirtazapine (open‐label)	15–45 mg	MADRS	Yes
		Ersoy/2008 [88]	21	22–69	Mirtazapine (open‐label)	7.5–30 mg	HDRS	Yes
Mianserin	Noradrenergic and serotonergic systems: Antagonist at 5‐HT2 receptors, enhances norepinephrine transmission [89].	Costa/1985 [90]	73	≥ 18	Mianserin/placebo	30–60 mg	HDRS/ZSRDS	Yes
		Heeringen/1996 [91]	55	≥ 18	Mianserin/placebo	30–60 mg	HRSD	Yes
Ketamine	Glutamatergic system: NMDA receptor antagonist, promotes synaptic plasticity and neurogenesis [92]. Also, neuroprotection [93].	Liu/2021 [94]	303	18–65	S‐ketamine/R‐ketamine/placebo	2 ml of 0.125 mg/kg	HDRS	Yes
Amitriptyline	Serotonergic and noradrenergic systems: Tricyclic antidepressant, inhibits reuptake of serotonin and norepinephrine [95, 96].	Pezzella/2001 [79]	175	18–65	Paroxetine/amitriptyline	75–150 mg	MADRS	Yes
Methylphenidate	Dopaminergic system: Increases dopamine availability, improves energy and mood [97].	Guan/2014 [98]	88	≥ 18	Mirtazapine+methylphenidate/mirtazapine+placebo	10–20 mg	MADRS	Yes
		Homsi/2001 [99]	41	30–90	Methylphenidate (open‐label)	10–20 mg	Direct question	Yes
		Sullivan/2017 [100]	35	≥ 18	SSRI+methylphenidate/SSRI+placebo	10–20 mg	MADRS/HADS	No
Psilocybin	Serotonergic system: Agonist at 5‐HT2A receptors, enhances neural plasticity and connectivity [101, 102, 103].	Ross/2016 [104]	29	22–75	Psilocybin/niacin	0.3 mg/kg	HADS	Yes
		Griffiths/2016 [105]	51	Not reported	Low‐dose psilocybin/high‐dose psilocybin	22 or 30 mg/70 kg	GRID‐HDRS	Yes
		Agrawal/2024 [106]	30	30–78	Psilocybin (open‐label)	25 mg	MADRS/HDRS	Yes
Diclofenac	Inflammatory system: NSAID, reduces inflammation that may contribute to depressive symptoms [107].	Mohammadinejad/2015 [83]	52	18–70	Celecoxib/diclofenac	100 mg	HDRS	Yes
Desipramine	Noradrenergic system: Tricyclic antidepressant, primarily inhibits norepinephrine reuptake [108].	Holland/1998 [75]	40	Not reported	Desipramine/fluoxetine	100–150 mg	HDRS	Yes
		Musselman/2006 [80]	35	18–75	Paroxetine/desipramine/placebo	50–175 mg	HDRS	No
Thioridazine	Dopaminergic system: Antagonist at dopamine receptors, mood‐stabilizing effects [109].	Johnston/1972 [110]	50	31–73	Thioridazine/placebo	75 mg	Physician ratings	Yes

Abbreviations: BZSDS, Brief Zung Self‐Rating Depression Scale; CES‐D, Center for Epidemiologic Studies Depression Scale; COX‐2, Cyclooxygenase‐2; HADS, Hospital Anxiety and Depression Scale; HDRS, Hamilton depression rating scale; MADRS, Montgomery‐Åsberg Depression Rating Scale; NMDA, N‐methyl‐D‐aspartate; NSAID, nonsteroidal anti‐inflammatory drug; POMS‐DD, Profile of Mood States—Depression‐Dejection subscale; SSRI, selective serotonin reuptake inhibitors.

### 4.1 Monoaminergic Antidepressants

#### 4.1.1 Fluoxetine

Fluoxetine is an SSRI antidepressant that increases serotonin levels and is used in the treatment of several psychiatric disorders, including MDD, obsessive‐compulsive disorder, and anxiety disorders [71, 72].

A study on patients with malignant tumors discovered the efficacy of a treatment with 20 mg/day of fluoxetine for 6 weeks in reducing depression [111]. A study investigated the effectiveness of fluoxetine in reducing depression in advanced cancer patients [73]. Participants received 20 mg of fluoxetine daily for 12 weeks, and results showed that depressive symptoms were significantly lower in the fluoxetine‐treated group compared to the control group. Another randomized clinical trial in 1998 administered 20 mg of fluoxetine daily to participants with cancer for 6 weeks, reporting a significant improvement in depression and anxiety [75]. Further systematic review studies support the previous findings regarding the efficacy of fluoxetine treatment [112, 113]. Additionally, Riblet et al. conducted a meta‐analysis and found fluoxetine to be superior to the placebo in the treatment of cancer‐related depression [114]. However, the Razavi et al. study demonstrates no clinically significant efficacy of 20 mg/day fluoxetine in relieving depression after 5 weeks of treatment, but it was more effective than a placebo in improving the global psychological adjustment [74].

Fluoxetine was found to be safe and well‐tolerated, with only a few side effects. Nausea, vomiting, and other digestive issues were more common in the fluoxetine group [73, 74]. Additionally, neuropsychiatric symptoms were frequently reported; however, their occurrence did not differ statistically significantly between the fluoxetine and placebo groups [74].

#### 4.1.2 Paroxetine

Paroxetine is another SSRI, widely used in the treatment of MDD, generalized anxiety disorder, and obsessive‐compulsive disorder [76, 77].

An animal study found that paroxetine improves depression in mice with colorectal cancer complicated and reduces inflammatory signaling [115]. To further assess the efficacy of paroxetine in cancer, an RCT examined the effect of paroxetine on depression in breast cancer patients receiving chemotherapy. Obtaining 20 mg/day of paroxetine lowered depression, but not fatigue, in patients who started paroxetine 7 days following the initial chemotherapy cycle and ending 7 days following the fourth one, compared to placebo [78]. Additionally, another eight‐week randomized clinical trial on breast cancer patients confirmed these results [79]; paroxetine was initially administered at 20 mg/day for 3 weeks. If clinically indicated, the dose could be adjusted to 40 mg/day at the end of Week 5. Consistent progress was observed throughout the eight‐week study period for both depressive symptoms and quality of life. Three other studies included the mentioned studies within their analyses and confirmed the effectiveness of paroxetine [112, 113, 114]. On the other hand, Musselman et al.'s study on breast cancer patients with major depression or adjustment disorder with depressed mood did not find any notable improvement in depression and anxiety in the paroxetine group compared to placebo after 6 weeks of treatment, and reported a high response rate in the placebo group [80]. A 2014 systematic review also referenced the previous study, highlighting the same effect of paroxetine compared to placebo [116].

In terms of safety and tolerability, no deaths were reported in the studies. However, one case of severe leukopenia occurred in the paroxetine group, which was considered unrelated to the medication. The most frequently observed side effects included nausea, leukopenia, headache, gastrointestinal (GI) discomfort, and insomnia [79]. While anticholinergic effects were less common, some participants experienced symptoms such as dry mouth and increased sweating. Somnolence was among the most common adverse experiences associated with the medication [78, 79].

#### 4.1.3 Amitriptyline

Amitriptyline is a TCA that acts mainly by inhibiting the reuptake of serotonin and norepinephrine. This medication is used in several psychiatric conditions, such as anxiety and MDD [95, 96].

A case study has shown that an injection of 10–75 mg of amitriptyline notably enhances the mood in terminally ill cancer patients with severe depression and a death wish [117]. A study mentioned earlier in the paroxetine section examined the application of 75 mg/day of amitriptyline to breast cancer patients for 3 weeks, followed by an increase in dose to 150 mg/day if indicated [79]. The depressive symptoms and quality of life were improved remarkably after 8 weeks of treatment. Furthermore, two systematic reviews supported these findings regarding the efficacy of amitriptyline on depression in cancer patients [112, 113]. However, another meta‐analysis evaluating efficacy reported no significant effect after 4 weeks of treatment with amitriptyline [114].

Amitriptyline has been evaluated for its safety and tolerability in an RCT, and three patients experienced serious but non‐fatal adverse events in the amitriptyline group, including severe pain, severe leukopenia, and moderate upper respiratory tract infection. However, these events were determined to be unrelated to the medication. Anticholinergic effects, mostly dry mouth and constipation, and nausea were the most common adverse effects of amitriptyline [79]. Other reported side effects included leukopenia, fatigue, GI issues, and somnolence. Among the treatment‐related side effects, somnolence and dry mouth were the most frequently observed.

#### 4.1.4 Desipramine

Desipramine is a TCA that functions as a relatively selective norepinephrine reuptake inhibitor and is primarily used in MDD treatment [108].

The effect of desipramine on depression in cancer patients was measured in a study conducted by Holland et al. in 1998 [75]. Participants who received 100–150 mg/day of desipramine showed significant improvement in depression, anxiety, and quality of life after a six‐week treatment course. In contrast, another study on breast cancer patients with major depression reported no significant progression in depressive symptoms after treatment with 50–175 mg/day of desipramine compared to the placebo [80]. A meta‐analysis also used the aforementioned study and reported no advantage of desipramine over placebo in cancer‐related depression [114].

The safety and tolerability of desipramine were assessed, and no deaths or serious adverse events were reported. The most common side effects included nausea, headache, diarrhea, anorexia, and somnolence [75].

The inconsistent findings for SSRIs like paroxetine and fluoxetine, as well as for TCAs, are notable and may reflect a fundamental mechanistic mismatch. These agents primarily target monoamine reuptake, a mechanism that does not directly address the core pathophysiological drivers of inflammation, HPA axis hyperactivity, or glutamate excitotoxicity prevalent in cancer‐related depression. The high placebo response rate noted in some trials [80] could suggest that non‐pharmacological factors, such as the enhanced psychosocial support inherent in trial participation, play a significant role. Alternatively, it may indicate that the biological heterogeneity of depression in this population is not adequately addressed by a single‐mechanism agent, leading to efficacy only in a subset of patients whose depression is less driven by inflammatory or glutamatergic pathways. This underscores the limitations of a purely monoamine‐based approach and reinforces the need for therapies targeting the specific neurobiology of this condition.

### 4.2 Tetracyclics

#### 4.2.1 Mirtazapine

Mirtazapine blocks presynaptic α_2_‐adrenergic auto‐receptors, which enhances the release of norepinephrine and serotonin [84]. Additionally, it antagonizes specific postsynaptic 5‐HT2 and 5‐HT3 receptors, promoting serotonergic activity while reducing side effects like nausea and sexual dysfunction [85, 118]. Mirtazapine is commonly used in the treatment of depression, anxiety, and insomnia [84, 119, 120].

Cankurtaran et al. studied the effectiveness of mirtazapine in cancer patients with major depression, anxiety, or adjustment disorder [86]. Patients were divided into three groups: mirtazapine, imipramine, and a control group. The mirtazapine group showed a significantly greater improvement in depression, anxiety, and insomnia compared to the other groups. These findings align with two different studies that reported a notable reduction in depression and anxiety after 1 week and 1 month of treatment with 15 mg of mirtazapine daily, with effects lasting until the end of the follow‐up [87, 88].

Alongside its therapeutic effects, the adverse effects of mirtazapine in cancer patients were examined. According to studies, mirtazapine is generally safe, and none of the RCTs reported the discontinuation of treatment due to the side effects [86, 87, 88]. Sedation and dizziness were among the most common adverse effects of mirtazapine; however, these side effects may occur initially and both diminish over time [87]. Weight gain was another common effect of mirtazapine, and it resulted in a significant increase in body weight compared to the placebo, which may be beneficial for cancer patients suffering from unintentional weight loss [86]. Other adverse effects mentioned in the studies were fatigue, hand tremor, and restless leg syndrome [88].

Mirtazapine's consistent efficacy may be attributable to its multi‐modal mechanism of action, which aligns well with the multifaceted pathophysiology of cancer‐related depression. Beyond its effects on noradrenergic and serotonergic systems, mirtazapine has been shown to rapidly attenuate HPA axis hyperactivity [121]. Studies demonstrate that it can significantly reduce cortisol secretion in depressed patients, often within the first week of treatment [122]. This direct pharmaco‐endocrinological effect on the HPA axis, one of the core mechanisms discussed in Section 3, distinguishes it from SSRIs and TCAs. By targeting both neurotransmitter systems and HPA axis dysregulation, mirtazapine may offer a more comprehensive treatment for the specific biological signature of depression in cancer patients. Furthermore, its known side effects of sedation and weight gain can be clinically advantageous in an oncology setting where insomnia and cachexia are common comorbidities.

#### 4.2.2 Mianserin

Mianserin is a second‐generation tetracyclic antidepressant [89]. It demonstrated antidepressant efficacy with minimal anticholinergic effects [89, 123].

The efficacy of treatment with mianserin in cancer patients with depression has been examined in several studies; a randomized clinical trial in 1985 found that women with cancer suffering from depression who received 30 mg/day mianserin for 1 week followed by an increased dose of 60 mg/day for 3 weeks showed significantly greater improvement in depression severity and duration compared to placebo [90]. Another RCT on breast cancer patients, using the same dosing regimen, reported similar antidepressant effects [91]. However, unlike the previous study, it found no significant difference in the anxiety‐somatization factor between the mianserin and placebo groups. In this regard, three recent systematic reviews also support the efficacy of mianserin in improving depression in cancer patients [112, 113, 116]. Also, Riblet et al. performed a meta‐analysis in 2014, which revealed a strong effect of mianserin in alleviating depression in cancer patients after 4 weeks of treatment [114].

Studies evaluating the safety and tolerability of mianserin found it to be generally safe, with no significant adverse effects compared to placebo. Mild drowsiness was the most common side effect, but it decreased with continued treatment [90, 91]. Postural symptoms, such as vertigo, were reported only in the mianserin group rather than the placebo group. Additionally, some patients experienced GI issues, including nausea, vomiting, and dry mouth [91].

### 4.3 Anti‐Inflammatories

#### 4.3.1 Celecoxib

Celecoxib is a selective COX‐2 inhibitor nonsteroidal anti‐inflammatory drug (NSAID) [81] that has shown potential as an adjunctive therapy in psychiatric disorders, particularly depression and schizophrenia [124, 125]. Research suggests that celecoxib may reduce depressive symptoms by targeting inflammation, a key factor in its etiopathology [61].

Several studies have investigated the effectiveness of celecoxib in managing depression among cancer patients. Alamdarsaravi et al. (2015) conducted a randomized clinical trial to evaluate the safety and efficacy of six‐week celecoxib monotherapy in colorectal cancer patients undergoing chemotherapy with mild to moderate depression [82]. The results indicated that 400 mg/day of celecoxib significantly improved depressive symptoms, increased response rates, and faster time to remission compared to placebo. These findings are consistent with Mohammadinejad et al.'s study on breast cancer patients, further supporting the effectiveness of 400 mg/day celecoxib in reducing depression severity regardless of remission [83].

The safety and tolerability of celecoxib were also assessed, and it was found to be well‐tolerated. Previous studies reported no serious adverse effects, and no cardiovascular events were detected either clinically or through electrocardiogram [82, 83]. Regarding GI complications, abdominal pain and diarrhea were the most commonly reported issues. However, the study by Alamdarsaravi et al. found no significant differences in GI complications between the celecoxib and placebo groups [82].

#### 4.3.2 Diclofenac

Diclofenac is an NSAID with a non‐selective COX inhibitory effect [107]. Recent studies suggest that it may have antidepressant and anxiolytic effects, possibly due to its role in modulating monoamine turnover and reducing inflammation [126].

A study by Mohammadinejad et al. on breast cancer patients, previously presented, also assessed the effect of 100 mg of diclofenac daily on mild to moderate depression [83]. The symptoms significantly improved after both 3 and 6 weeks of treatment, although complete remission was not achieved. Although diclofenac is retained here for completeness because it met the inclusion criteria, the available evidence is limited; therefore, its potential antidepressant effect should be interpreted cautiously.

The safety and tolerability of diclofenac were evaluated in the studies. No serious adverse effects or deaths were reported. The most common side effects were GI issues, including discomfort, abdominal pain, nausea, vomiting, and diarrhea. In addition, no cardiovascular events were detected through electrocardiogram or physical examination [83].

The promising findings for the selective COX‐2 inhibitors provide promising but preliminary clinical validation for the inflammatory hypothesis of cancer‐related depression outlined in Section 3.1.2. By targeting the upstream production of pro‐inflammatory prostaglandins, these agents demonstrate that modulating the immune response can be an effective antidepressant strategy in this population, moving beyond traditional neurotransmitter‐focused approaches.

### 4.4 Glutamatergic/Psychodelics

#### 4.4.1 Ketamine

Ketamine is an uncompetitive NMDA receptor antagonist originally developed as an anesthetic and has recently gained interest for its rapid‐acting antidepressant effects, especially in treatment‐resistant depression [92].

A case report in 2018 found that continuous infusion of 0.2 mg/kg/h of ketamine alleviates the depressive symptoms of a cancer patient with severe depression [127]. Tomy et al. in a case series reported the significant reduction of depressive symptoms and suicidal ideation in cancer patients with treatment‐resistant depression following the administration of intranasal S‐ketamine, and the effect was sustained for up to a year [128]. However, the effect was temporary. Moreover, a recent study investigated the effect of pre‐treatment with ketamine on postoperative depression in breast cancer patients with mild to moderate depression [94]. An injection of 2 mL of 0.125 mg/kg before surgery significantly reduced short‐term postoperative depression compared to the control group. Additionally, S‐ketamine was more effective than racemic ketamine in reducing depression. An open‐label study evaluated the effect of intranasal racemic ketamine (50–150 mg) in patients with advanced cancer. Results indicated a rapid antidepressant effect that lasted for a week [129]. Similarly, a systematic review was conducted, identifying five studies on the effect of racemic or S‐ketamine on depression in cancer patients. The results of all the included studies indicated that a single intraoperative dose of ketamine with a dosage of 0.1–0.5 mg/kg significantly improved postoperative depressive symptoms rapidly, with S‐ketamine being more effective than racemic ketamine [130].

Regarding the adverse effects, findings support the safety profile of ketamine without any added perioperative risk. The studies measured the intraoperative and postoperative complications, and there was no difference between the S‐ketamine and racemic ketamine groups and the control group [94, 130]. Intranasal administration of the drug has some adverse effects, with dysgeusia being the most common, followed by fatigue, dissociation, nausea, and headaches. All adverse effects were mild and transient, resolving within 2 h post‐administration [129].

The rapid and robust antidepressant effects of the uncompetitive NMDA receptor antagonist ketamine provide direct clinical validation for the glutamate excitotoxicity pathway, a key mechanism discussed in Section 3.1.2. Its efficacy likely stems from its ability to counteract the effects of endogenous NMDA agonists like QUIN, which are upregulated as a direct consequence of the inflammation‐driven IDO pathway. By blocking NMDA receptors, ketamine effectively interrupts the final common pathway of this neurotoxic cascade. Additionally, it has demonstrated a neuroprotective role in reducing neuroinflammation and enhancing neuroplasticity in a recent study using in vitro stress models [93]. These provide a clear mechanistic rationale for its utility in treatment‐resistant cases, where traditional antidepressants that do not target this glutamatergic pathway may fail.

#### 4.4.2 Psilocybin

Psilocybin is a tryptamine serotonergic psychedelic and 5‐HT2A agonist that has antidepressant effects after a single dose and is also used in anxiety and post‐traumatic stress disorder treatment [101, 102, 103].

Recent clinical trials have focused on the efficacy of psilocybin in treating depression in cancer patients. A single dose of 0.3 mg/kg psilocybin combined with psychotherapy led to rapid, significant, and long‐lasting improvements in anxiety and depression, with effects sustained at a 6.5‐month follow‐up [104]. Additionally, existential distress significantly decreased, while quality of life showed a notable improvement. These findings are supported by another randomized crossover clinical trial that investigated the efficacy of a high dose of psilocybin (22 or 30 mg/70 kg) for depression in patients with a life‐threatening cancer [105]. The results indicated a significant decrease in depression, anxiety, and mood disturbance, sustained at a six‐month follow‐up, along with increased life satisfaction and quality of life compared to the low‐dose group. Similarly, a study by Agrawal et al. aligns with these findings, showing a clinically meaningful sustained reduction in depression and anxiety following treatment with a 25 mg dose of psilocybin [106].

Regarding the safety of the medication, no serious adverse effects were observed. Griffiths et al. study adjusted the psilocybin dose from 30 to 22 mg/70 kg after the dropout of two patients [105]. All reported side effects were mild, tolerable, and transient, resolving completely by the end of the session. The most common medical side effects included a temporary moderate increase in blood pressure and heart rate, nausea or vomiting, headaches, and physical discomfort [104, 105]. Psychological side effects, such as brief episodes of anxiety and transient near‐psychotic symptoms, were also reported. No persistent hallucinogen perception disorder or continuous psychosis was noted [104, 105].

Psilocybin, a potent serotonin 5‐HT2A receptor agonist, offers a novel paradigm. Rather than simply modulating synaptic neurotransmitter levels, its mechanism is thought to induce rapid and lasting changes in neuroplasticity and functional brain connectivity. This may serve to reset dysfunctional neural circuits that have become pathologically rigid due to chronic stress, inflammation, and existential distress, explaining its profound and sustained effects on both depression and anxiety in cancer patients.

### 4.5 Stimulants

#### 4.5.1 Methylphenidate

Methylphenidate is a central nervous system stimulant that increases the levels of norepinephrine and dopamine in the brain by blocking their reuptake. It is primarily used to treat attention‐deficit hyperactivity disorder and improve focus, attention, and impulse control [97].

In a study on hospitalized cancer patients with depression, participants received psychostimulants, including an average dose of 8 mg/day of methylphenidate. A rapid improvement in depressive symptoms was reported [131]. The addition of methylphenidate to mirtazapine in terminally ill cancer patients with MDD was studied in a four‐week randomized clinical trial [98]. Participants initially received either 10 mg/day of methylphenidate, with an optional additional dose from Day 3 if needed, or a placebo. The results indicated that adding methylphenidate to standard depression treatment enhances antidepressant effects, showing a notable improvement by the third day and a significant clinical response from the second week onward. In addition, an open‐label study on advanced cancer patients with depression found a significant improvement in depressed mood after treatment with 10 mg/day of methylphenidate [99]. In contrast, another study reported no significant effect of methylphenidate in SSRI‐treated advanced cancer patients after 18 days of trial with the same treatment protocol [100].

The safety and resilience of this medication were also assessed. Symptoms were generally mild, and no adverse effect‐related deaths were reported. Neurological involvements, including psychosis, seizure, and tremor, were more frequent in the methylphenidate group than in the placebo group. Stomach complaints, nausea, and vomiting were also reported among the methylphenidate group [98].

The utility of methylphenidate in this context is best understood through the lens of inflammation‐induced sickness behavior. As previously discussed, pro‐inflammatory cytokines can induce symptoms of fatigue, anhedonia, and psychomotor retardation that overlap significantly with depression. This syndrome is hypothesized to be mediated, in part, by inflammation‐induced dysfunction in dopaminergic signaling within the basal ganglia, a key brain region for motivation and motor control. Methylphenidate, a dopamine and norepinephrine reuptake inhibitor, directly counteracts this specific deficit by enhancing dopaminergic transmission. Therefore, its efficacy is likely not a general antidepressant effect but rather a targeted reversal of the motivational and energetic deficits driven by the underlying inflammatory process, making it particularly useful for patients in whom fatigue and apathy are prominent symptoms.

### 4.6 Dopamine Antagonists

#### 4.6.1 Thioridazine

Thioridazine is a first‐generation antipsychotic medication. It is a dopamine D1 and D2 receptor antagonist that is used in the treatment of schizophrenia [109].

A randomized clinical trial in 1972 studied the effect of the addition of 75 mg daily of thioridazine in terminal cancer patients with depression [110]. The study found that after 1 week of treatment, patients showed an improvement in depressed mood compared to the placebo group. However, this effect was not sustained, as no significant difference was observed between the groups at three and 6 weeks of treatment. Thioridazine is included for completeness because it met the inclusion criteria; however, its clinical relevance is constrained by the age of the available evidence and the limited evidence.

The safety and tolerability of thioridazine were assessed. No adverse effects were pointed out or documented during the study, and the medication was well tolerated [110]. However, it has been withdrawn from many markets around the globe as it increases the risk of arrhythmia from QT‐prolongation [132], and the clinical trial that supports the use of this drug can be considered outdated.

## 5 Limitations

This study has some limitations, including the lack of a rigorous systematic literature search. This review provides a formal narrative synthesis but is not a systematic review. Narrative reviews can offer a broader perspective and integration of mechanistic and clinical evidence but inherently use selective searches. A formal protocol or meta‐analytic methods were not used, so some relevant studies might be missed. It was attempted to mitigate bias by transparently describing the search and inclusion criteria.

Furthermore, the included studies exhibited certain limitations, such as small sample sizes, relatively short follow‐up periods, absence of post‐treatment monitoring, and a focus on statistical rather than clinical significance, which warrant careful consideration when interpreting the findings discussed herein. A primary concern is the prevalence of small sample sizes in many of the cited clinical trials. Studies with small samples often suffer from low statistical power, which has two insidious consequences. First, it reduces the probability of detecting a true therapeutic effect. Second, and more critically, it reduces the likelihood that a statistically significant finding actually represents a true effect while also leading to an overestimation of the magnitude of that effect. The average statistical power in neuroscience and related fields is estimated to be low, suggesting that some of the positive findings reported in the literature may be less reliable or reproducible than they appear [133]. Therefore, the evidence for many agents must be considered preliminary until confirmed in larger, adequately powered trials.

Another significant limitation of the existing literature is the relatively short follow‐up duration of most trials. This is problematic because it may fail to capture the full, long‐term benefits of effective depression treatment and reveal late‐emerging risks. These studies may miss enduring effects that appear well after treatment ends, sometimes referred to as legacy effects [134].

Also, diclofenac and thioridazine are supported by limited and historically dated evidence; accordingly, their inclusion should be interpreted as descriptive rather than as evidence of robust clinical utility.

Additionally, some included trials are relatively old, and changes in diagnostic criteria and oncology treatments may limit their applicability to current clinical practice.

## 6 Ethical Considerations in Pharmacotherapy

The treatment and study of depression in cancer patients are fraught with complex ethical challenges, primarily revolving around the principles of informed consent, autonomy, and beneficence. Patients with cancer and comorbid depression represent a uniquely vulnerable population. The psychological burden of both diagnoses can significantly impair decisional capacity, including the ability to understand, appreciate, and reason about treatment options and their consequences. Symptoms like hopelessness, anhedonia, and cognitive deficits can cloud judgment, making it difficult for patients to make choices that align with their authentic values. Clinicians must therefore navigate a delicate balance: respecting patient autonomy while fulfilling their duty of beneficence by advocating for treatments that can alleviate suffering. This requires a rigorous yet compassionate assessment of decisional capacity, ensuring that a refusal of treatment is not merely a symptom of a treatable depressive disorder [135, 136, 137].

These ethical considerations are amplified in the context of clinical research. The inherent vulnerability of this population increases the risk of therapeutic misconception, that is, the tendency for participants to conflate the aims of research with personalized clinical care, potentially underestimating risks and overestimating direct personal benefits. Institutional review boards and researchers have a heightened responsibility to ensure that the informed consent process is robust, transparent, and continuous. This includes providing comprehensive psychosocial support not just as an adjunct, but as an ethical necessity to bolster patient understanding, mitigate distress, and safeguard true voluntarism throughout the trial. Ultimately, ethical practices in both clinical and research settings demand a patient‐centered approach that protects and empowers this vulnerable group [138, 139, 140].

## 7 Clinical Implications

The evidence synthesized in this review has several direct and actionable implications for clinicians managing depression in patients with cancer. The central takeaway is the necessity of shifting from a conventional, monoamine‐centric treatment algorithm to a more nuanced, mechanism‐informed strategy that aligns pharmacotherapy with the patient's specific biological and clinical presentation. Table 2 summarizes a conceptual mapping between common clinical proxies, the hypothesized dominant mechanism, and candidate pharmacotherapies. This schema is intended as a heuristic guide rather than a treatment algorithm.

**TABLE 2:** Conceptual mapping of clinical proxies, hypothesized mechanisms, and candidate pharmacotherapies in cancer‐related depression.

Clinical proxy/marker	Hypothesized dominant mechanism	Candidate pharmacotherapy	Brief rationale
Elevated C‐reactive protein, Interleukin 6, inflammatory phenotype	Inflammation‐driven depression [28, 61]	Celecoxib [82]	May be useful when inflammatory burden appears prominent
Prominent fatigue, psychomotor slowing, low motivation, anhedonia	Dopaminergic deficit/inflammatory overlap	Methylphenidate [98, 141]	May help when fatigue and slowing dominate the clinical picture
Severe treatment‐resistant depression, need for rapid symptom relief	Glutamatergic dysregulation [52, 142]	Ketamine	Biologically plausible for rapid‐acting antidepressant effect
Insomnia, anorexia, weight loss, poor appetite	Arousal/appetite dysregulation; mixed serotonergic and noradrenergic profile	Mirtazapine [86]	May be helpful when sleep and appetite symptoms are prominent
Mixed depressive symptoms with pain/neuropathic burden and suspected inflammatory contribution	Inflammation plus somatic symptom burden [28, 61]	Celecoxib or other adjunctive strategy [61, 82]	Can be considered as an adjunct in selected patients
Chronic stress phenotype, prolonged illness burden, hyperarousal [69, 86]	Hypothalamic–pituitary–adrenal axis dysregulation [29]	Symptom‐guided antidepressant selection	A conceptual link rather than a direct biomarker‐based indication

*Note:* Importantly, this table is presented as a conceptual framework to support individualized reasoning in cancer‐related depression; it should not be interpreted as a validated decision rule or a substitute for clinical judgment.

The inconsistent efficacy of traditional SSRIs and TCAs should not be viewed as treatment failure alone, but rather as a predictable outcome of a mechanistic mismatch. Clinicians can use this framework to set realistic expectations and justify moving more quickly to agents with broader mechanisms of action. For instance, atypical antidepressants like mirtazapine, which demonstrate effects on the HPA axis in addition to its neurotransmitter modulation, may be considered a more robust first‐ or second‐line option, particularly in patients presenting with comorbid insomnia and anorexia.

Also, the patient's symptom profile should guide treatment selection beyond a simple depression diagnosis. For patients in whom fatigue, anhedonia, and psychomotor retardation are the most prominent features, psychostimulants such as methylphenidate may offer a more targeted and rapid‐acting intervention than traditional antidepressants by directly addressing underlying dopaminergic deficits.

Additionally, this review provides a strong rationale for considering agents that target inflammation and glutamate pathways, especially in patients who do not respond to initial treatments. In clinical settings where inflammatory markers are available or where a high inflammatory state is suspected, adjunctive use of anti‐inflammatory agents like celecoxib is a viable, evidence‐based strategy. Similarly, for patients with severe or treatment‐resistant depression, the rapid and potent effects of the NMDA receptor antagonist ketamine offer a mechanistically sound therapeutic option that directly counteracts the neurotoxic effects of the inflammation‐driven KYN pathway.

Furthermore, this mechanism‐based framework encourages a paradigm shift towards personalized psycho‐oncology. By conceptualizing cancer‐related depression as a heterogeneous condition driven by a cascade of inflammation, HPA axis hyperactivity, and glutamate excitotoxicity, clinicians can make more rational and targeted pharmacotherapeutic choices, potentially improving treatment outcomes and enhancing the quality of life for their patients.

Ultimately, it should be considered that patients with cancer frequently receive complex pharmacological regimens, increasing the risk of clinically significant drug–drug interactions. Notably, SSRIs like fluoxetine and paroxetine are potent inhibitors of cytochrome P450 2D6 (CYP2D6), which may reduce the bioactivation of tamoxifen and potentially compromise its therapeutic efficacy in breast cancer. However, recent population data found no difference in survival for women taking tamoxifen with paroxetine/fluoxetine versus other antidepressants [143, 144]. Nonetheless, considering alternatives (e.g., citalopram) is suggested when tamoxifen is used, and careful selection of antidepressants with lower interaction potential is essential in line with oncologic guidelines.

## 8 Conclusion

Depression in patients with cancer is a multifactorial comorbidity involving a distinct neurobiological signature characterized by inflammation, HPA axis dysregulation, and glutamate excitotoxicity.

This review of the evidence suggests that a mechanism‐informed approach to pharmacotherapy is superior to a conventional, monoamine‐based strategy. The mechanism‐informed perspective represents a shift from traditional symptom‐based treatment models toward biologically grounded, personalized strategies. Interventions that directly target these underlying pathways, such as the anti‐inflammatory agent celecoxib, the glutamate modulator ketamine, and atypical antidepressants like mirtazapine and mianserin, demonstrate more consistent and robust efficacy. Emerging therapies like psilocybin offer a novel paradigm by promoting neuroplasticity. In contrast, the conflicting results for traditional SSRIs and TCAs likely reflect their inability to adequately address this complex pathophysiology. Other agents such as diclofenac, methylphenidate, and thioridazine show utility in specific contexts. Therefore, a paradigm shift is needed in the clinical management of cancer‐related depression, moving away from a trial‐and‐error approach toward a personalized strategy guided by biological mechanisms.

Future research should prioritize neuroimaging and biomarker‐stratified trials to translate this mechanism‐based framework into personalized clinical practice. For example, studies could use inflammatory markers such as C‐reactive protein or IL‐6 to identify patients most likely to respond to anti‐inflammatory strategies (e.g., celecoxib), or use markers of KYN pathway activation to predict response to glutamate modulators like ketamine. Such an approach represents the critical next step in optimizing therapeutic outcomes and improving the quality of life for this vulnerable population.

## Author Contributions

S.H.: data curation, supervision, writing – original draft, writing – review and editing. K.F.: data curation, writing – original draft. K.K.: data curation, writing – original draft; G.H.: writing – original draft. A.S.: conceptualization, project administration, supervision, data curation, writing – original draft, writing – review and editing. All authors approved the final version of the manuscript and had accountability for all work aspects.

## Funding

The authors have nothing to report.

## Ethics Statement

The authors have nothing to report.

## Consent

The authors have nothing to report.

## Conflicts of Interest

The authors declare no conflicts of interest.

## Data Availability

Data sharing not applicable to this article as no datasets were generated or analysed during the current study.
